# Impact of Chronic Prenatal Stress on Maternal Neuroendocrine Function and Embryo and Placenta Development During Early-to-Mid-Pregnancy in Mice

**DOI:** 10.3389/fphys.2022.886298

**Published:** 2022-06-13

**Authors:** Neta Gotlieb, Kathryn Wilsterman, Samantha L. Finn, Madison F. Browne, Savannah R. Bever, Eiko Iwakoshi-Ukena, Kazuyoshi Ukena, George E. Bentley, Lance J. Kriegsfeld

**Affiliations:** ^1^ Department of Psychology, University of California, Berkeley, Berkeley, CA, United States; ^2^ Department of Integrative Biology, University of California, Berkeley, Berkeley, CA, United States; ^3^ Division of Biological Sciences, University of Montana, Missoula, MT, United States; ^4^ Biology Department, Colorado State University, Fort Collins, CO, United States; ^5^ Laboratory of Neurometabolism, Graduate School of Integrated Sciences for Life, Hiroshima University, Hiroshima, Japan; ^6^ Helen Wills Neuroscience Institute, University of California, Berkeley, Berkeley, CA, United States

**Keywords:** RFRP-3, prolactin, glucocorticoids, progesterone, pituitary lactotrophs, GnIH, TIDA neurons

## Abstract

Psychological stress, both leading up to and during pregnancy, is associated with increased risk for negative pregnancy outcomes. Although the neuroendocrine circuits that link the stress response to reduced sexual motivation and mating are well-described, the specific pathways by which stress negatively impacts gestational outcomes remain unclear. Using a mouse model of chronic psychological stress during pregnancy, we investigated 1) how chronic exposure to stress during gestation impacts maternal reproductive neuroendocrine circuitry, and 2) whether stress alters developmental outcomes for the fetus or placenta by mid-pregnancy. Focusing on the stress-responsive neuropeptide RFRP-3, we identified novel contacts between RFRP-3-immunoreactive (RFRP-3-ir) cells and tuberoinfundibular dopaminergic neurons in the arcuate nucleus, thus providing a potential pathway linking the neuroendocrine stress response directly to pituitary prolactin production and release. However, neither of these cell populations nor circulating levels of pituitary hormones were affected by chronic stress. Conversely, circulating levels of steroid hormones relevant to gestational outcomes (progesterone and corticosterone) were altered in chronically-stressed dams across gestation, and those dams were qualitatively more likely to experience delays in fetal development. Together, these findings suggest that, up until at least mid-pregnancy, mothers appear to be relatively resilient to the effects of elevated glucocorticoids on reproductive neuroendocrine system function. We conclude that understanding how chronic psychological stress impacts reproductive outcomes will require understanding individual susceptibility and identifying reliable neuroendocrine changes resulting from gestational stress.

## Introduction

In women, maternal stress, including psychological stress such as anxiety and depression, is associated with an increased risk of miscarriage, preterm birth, and low birth weight, particularly if stress occurs during the first trimester of pregnancy (Lou et al., 1994; Neugebauer et al., 1996; Hobel et al., 1999; Paarlberg et al., 1999; Kurki et al., 2000; Glover, 2014; Sheriff et al., 2017; Van den Bergh et al., 2017; Valsamakis et al., 2019). In animal models, stress exposure similarly results in higher risk for negative pregnancy outcomes, including embryo resorption, reduced litter size, and intrauterine growth restriction (Lesage et al., 2004; Paternain et al., 2013; Jafari et al., 2017). Understanding the neuroendocrine mechanisms by which stress increases negative pregnancy outcomes is important for identifying clinical targets to treat or mitigate these effects in both humans and animals.

Activity of the hypothalamic-pituitary-adrenal (HPA) axis is a central component of the physiological stress response, and its excess activity has been linked to negative effects on reproductive outcomes. When the HPA axis is activated, the adrenal gland increases the production and release of glucocorticoids (i.e., cortisol in humans and corticosterone in mice) that can dysregulate the maternal hypothalamo-pituitary-gonadal (HPG) axis via direct and indirect actions on the hypothalamus and pituitary (Whirledge and Cidlowski, 2013, 2017). The RFamide-related peptide-3 (RFRP-3) system is a direct target of glucocorticoids and has been consistently implicated in the stress responses in rodents (Kirby et al., 2009; Geraghty et al., 2015; Yang et al., 2018; Singh et al., 2022). Most relevant to the present study, stress prior to gestation increases activity of the inhibitory RFRP-3 system, ultimately leading to reduced reproductive success (Geraghty et al., 2015). Dysregulation of the HPG axis via HPA activation can also suppress progesterone production, a steroid hormone critical for pregnancy maintenance (van Niekerk and Morgenthal, 1982; Wiebold et al., 1986; Parker and Douglas, 2010; Kajaysri and Nokkaew, 2014; Wilsterman et al., 2018), and modify pituitary prolactin secretion, a peptide hormone for which secretion pattern can predict poor pregnancy outcomes (Miller et al., 2004; Poletini et al., 2007).

Although the impact of HPA axis activation on reproductive function has been well-studied during non-pregnant physiological states, the interactions between these axes during gestation are not well characterized. Our previous work has shown that, in mice, chronic psychological stress inhibits ovarian progesterone synthesis in early pregnancy and this inhibition is associated with elevated corticosterone concentrations in maternal circulation (Wilsterman et al., 2018). However, the impact of chronic stress on hypothalamic neuroendocrine circuits critical for pregnancy maintenance and, how chronic stress affects embryonic and placental development remains to be fully elucidated. Thus, in the present study, we aimed to answer two broad questions: 1) How do neuroendocrine circuits critical for successful pregnancy maintenance and offspring development respond to chronic psychological stress during gestation, and 2) Is psychological stress during early pregnancy associated with altered embryonic and placental development?

To answer these questions, we compared multiple levels of the HPG axis in mouse dams subjected to daily restraint and predator odor stress beginning on day 1 of pregnancy to that of unstressed dams at three time points across gestation (day 5, 10, and 15). We also quantified the effects of stress exposure on embryonic and placental development. Within reproductive neuroendocrine circuits, we focused on potential interactions between hypothalamic RFRP-3 cells and tuberoinfundibular dopaminergic (TIDA) neurons that regulate pituitary prolactin secretion (Fuxe, 1964; Ben-Jonathan and Hnasko, 2001; Parker et al., 2011; Lyons et al., 2012). We predicted that, as with stress that occurs prior to pregnancy (Geraghty et al., 2015), stress during pregnancy would alter the RFRP-3 neuronal system and potentially its projections onto TIDA neurons, and thus gestational stress would impair reproductive success and fetal development. In addition, we quantified changes to developmental outcomes by examining fetal development, placental structure, and placental gene expression. We focused our gene expression analyses on six key genes that have been previously implicated in glucocorticoid regulation within the placenta [*GR*, *11βHSD1*, and *11βHSD2*; e.g., (Low et al., 1994; Jamieson et al., 1995)], placental responses to stress and fetal growth [*Phlda2*; (Tunster et al., 2014, 2; Janssen et al., 2016, 2; Tunster et al., 2016, 2)], or placenta-specific cell type differentiation or function [*Tpbpa* and *PLII*; e.g., (Faria et al., 1990; Faria and Soares, 1991; Hu and Cross, 2011; Albers et al., 2019)].

## Materials and Methods

### Animals

Adult (10–12 week old) C57BL/6J mice were purchased from the Jackson Laboratory (Sacramento, CA) and housed in ventilated cages on a 14:10 light/dark cycle (lights on at 07:00, lights off at 21:00) with laboratory chow and water available *ad libitum*. Humidity and temperature were held constant at 40% and 21°C, respectively. Experimental females were pair-housed with an age-matched male throughout the experiment. All animals were allowed to acclimate to the housing conditions for at least 1 week prior to commencing the experiment. All protocols were approved by the UC Berkeley Office of Laboratory Animal Care and were consistent with NIH guidelines for the care and use of laboratory animals.

### Experimental Procedures

**FIGURE 1:**
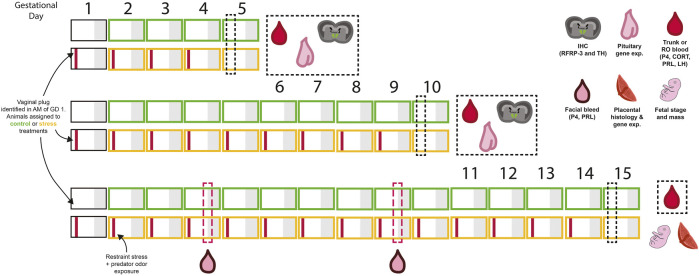
A summary of the experimental design and sampling timeline used in this experiment. Each box represents 1 day, with grey shading representing the dark period. Animals were assigned to either control (green) or stress (yellow) treatment groups on the day vaginal plugs were identified (gestational day 1). A combination of restraint and predator odor were used as the psychological stressor. Mice were exposed to the stressor in the mornings on each experimental day (indicated by the red bar within each box). Tissues were collected in the morning on days 5, 10, or 15 of pregnancy and tissues for further analysis, as indicated by cartoon symbols in the figure. For animals sampled on day 15 of gestation, evening blood samples were collected on days 4 and 9 to examine the evening prolactin surge. Refer to methods and results text for further details.

Experimental procedures and timeline are outlined in Figure 1. Virgin female mice were paired with sexually-experienced males and examined for vaginal copulatory plugs every morning. The morning a plug was observed was considered gestational day 1. Females were randomly assigned to chronic stress or control (non-stressed) groups. All females were massed each morning prior to treatment. Animals assigned to the chronic psychological stress group were moved each morning, beginning on day 1, to a separate room where they were restrained in a modified 50 ml centrifuge tube as previously described (Wilsterman et al., 2018). “Psychological stress” is used here to distinguish a perceived threat from a physical or physiological stressor. In addition, 15 µL of predator odor (undiluted fox urine, Minnesota Trapline, Inc; Pannock, MN) was applied to a fresh cotton ball and placed in the cage with each mouse during restraint. Daily stress exposure lasted 4 h from 09:00 to 13:00 (i.e., starting 2 h after lights on). Stress exposure was repeated daily until euthanasia. Non-stressed females remained in their home cages.

Females were euthanized during early pregnancy (day 5) through mid-pregnancy (day 10 and day 15) *via* intraperitoneal injection of sodium pentobarbital (200 mg/kg) followed by rapid decapitation or perfusion (see Figure 1 for timeline). Stress exposure did not occur on the morning of tissue collection. All blood samples were collected in fewer than 3 min from lifting the cage either via retro-orbital sinus bleeds (prior to perfusion, *N* = 6/group) or directly from the trunk after decapitation (*n* = 9-11/group). Blood was clotted at room temperature for 90 min prior to centrifugation at 1300 × g for 15 min at 4°C. Plasma was aspirated and then centrifuged a second time for 1 min to remove any residual red blood cells prior to aliquoting for storage.

Animals were perfused transcardially with 0.9% saline (2 min at 10 ml/min) followed by 4% paraformaldehyde (PFA; 5 min at 10 ml/min). After dissections, brains from perfused animals were submerged in PFA at 4°C for 3 h followed by cryoprotection using 30% sucrose (overnight at 4°C) prior to snap-freezing.

On day 15, the number of developing embryos in each uterine horn was counted and fetal developmental abnormalities (as detailed below) or resorption sites were recorded by an observer unaware of treatment conditions. The fetuses were individually massed and a subset (*n* = 4, 6 for control and stress, respectively) were assessed for developmental stage based on Theiler Staging (Theiler, 2013) by two independent observers unaware of experimental treatment. Placentae and pituitaries were flash frozen in isopentane on dry ice. Maternal hypothalami were dissected from the brain and divided in half along the midline before flash freezing. One half of each hypothalamus was used for RFRP-3 peptide quantification using an ELISA.

All tissues were stored at −80°C until assay and analysis.

### Brain Tissue Histology, Microscopy, and Image Analysis

#### Brain Immunohistochemistry

All immunohistochemistry was performed on brains fixed *via* perfusion with 4% PFA. Brains were sectioned in the coronal plane at 40 μm on a Leica 3050S cryostat and stored at −20°C in antifreeze solution until immunohistochemistry (IHC) was performed. To visualize RFRP-3-immunoreactive (RFRP-3-ir) cell bodies, their processes, and TIDA neurons, double-label immunofluorescence was performed on every fourth 40 μm brain section. To visualize TIDA neurons, an anti-tyrosine hydroxylase (TH) antibody was used, as TH is the rate-limiting enzyme in the conversion of tyrosine to dopamine. Briefly, free floating sections were washed in phosphate-buffered saline (PBS), incubated for 10 min in 0.5% hydrogen peroxide, washed in PBS again, and then blocked for 1 h in 3% normal goat serum suspended in PBS containing 0.1% Triton X-100 (PBT). Sections were then incubated for 48 h at 4°C in a rabbit polyclonal anti-RFRP-3 antibody (1:120,000; PAC 123/124) with 2% normal goat serum in PBT. After incubation in the primary antibody, sections were washed in PBT, incubated for 1 h in biotinylated goat anti-rabbit IgG (1:300, Vector Laboratories, Burlingame, CA), washed in PBT, and incubated for 1 h in avidin-biotin-horseradish peroxidase complex (ABC Elite Kit, Vector Laboratories). Sections were then washed with PBT followed by 0.6% biotinylated tyramide solution for 30 min. After washing with PBS, cells were labeled with the fluorophore, CY-2 streptavidin conjugate (1:150, Jackson ImmunoResearch Laboratories, West Grove, PA). Next, sections were washed with PBS and incubated for 1 h with 3% normal donkey serum suspended in PBT prior to being incubated for 48 h at 4°C in a mouse polyclonal anti-TH antibody (1:10,000; T2928, Sigma-Aldrich, St. Louis, MO) with 2% normal goat serum in PBT. Following incubation, sections were washed and labeled with the fluorophore, CY-3 donkey-anti-mouse (1:150, Jackson ImmunoResearch Laboratories, West Grove, PA). Sections were then incubated with DAPI (1:20,000, Invitrogen, catalog #D1306) for 10 min. Finally, sections were washed with PBS and mounted onto gelatin-coated slides, dehydrated and cleared with xylene, and coverslips were applied.

#### RFRP-3-ir and TIDA Neuron Image Analysis

To determine the number of RFRP-3-ir cells labeled and the percentage of TIDA neurons with close apposition from RFRP-3-ir fibers, sections were examined using conventional microscopy and the standard wavelengths for CY-2 (488 nm) and CY-3 (568 nm) with a Zeiss Z1 microscope (Thornwood, NY). Every fourth section through the dorsomedial hypothalamus was examined for RFRP-3-ir cell bodies. Every fourth section through the arcuate nucleus of the hypothalamus was examined for TH-ir and RFRP-3-ir terminal fibers. Photomicrographs were taken at 400×. Each label was captured in an automated Z-stack at 0.5-μm increments. Images were quantified in Fiji (Schindelin et al., 2012) by two investigators unaware of experimental treatment. The average counts per section were used for analyses. For each animal, the rostral-caudal extent of the arcuate nucleus was examined and three consecutive sections in the sequence were chosen based on TH-positive cell location (Supplementary Figure S1).

### Hormone Assays

All AM blood samples used in hormone analyses were collected from the trunk following rapid decapitation. All PM blood samples used in hormone analyses were collected via retro-orbital sinus bleeds. Progesterone was quantified using a Cayman Chemical Progesterone ELISA (Item No. 582601, Ann Arbor, MI). Intra- and inter-assay coefficients of variation (COV) for progesterone were 3.6 and 6.8%, respectively. Baseline corticosterone was quantified using an Enzo corticosterone ELISA kit (ADI-900-097; Enzo Life Sciences, Inc., Farmingdale, NY) according to the manufacturer’s protocol for small sample volumes. Intra- and inter-assay COV were 6.19 and 5.08%, respectively. Prolactin was assayed using a mouse prolactin ELISA kit from Abcam (ab100736, Cambridge, MA). Intra- and inter-assay COV were 3.6 and 4.2%, respectively. LH concentrations were quantified using LH ELISA, modified from (Ancel et al., 2012). The protocol was kindly provided by Dr. Jens D. Mikkelsen (Copenhagen University Hospital, Denmark). Briefly, 96-well microtiter plates were coated with 50 μL of bovine LHβ 518B7 monoclonal antibody (kindly provided by Lillian E Sibley, UC Davis) and incubated overnight at 4°C. Excess antibody was removed, and the plates were washed with 200 μL/well of 10 mM PBS with 0.1% Tween 20. The plates were blocked using 5% skim milk powder in PBS with 0.1% Tween 20 and incubated for 1 h at room temperature. Following washes, 50 μL of sample or standards of mouse LH (mouse RIA kit, National Hormone and Pituitary program, University of California, Harbor Medical Center, Los Angeles, CA), diluted in assay buffer, were added per well in duplicates and incubated for 2 h at room temperature. The plates were washed and 50 μL of rabbit polyclonal LH antibody (AFP240580Rb, National Hormone and Pituitary program, University of California, Harbor Medical Center, Los Angeles, CA) were added into each well and then incubated at room temperature for 90 min. After washing, 50 µL of a polyclonal goat anti-rabbit IgG conjugated to horseradish peroxidase (DAKO Cytomation, catalog #P0448) were added at 1:2000 dilution and incubated for 1 h at room temperature. After washing, 100 μL of o-phenylenediamine [OPD (Invitrogen, catalog # 00–2003)] in citrate buffer were added to all the wells. The color reaction was allowed to develop for 30 min in the dark. The reaction was stopped by adding 50 μL of 3M HCl per well and the optical density of each well was read immediately at 490 nm with a correction using at 655 nm. Samples which did not reach the limit for detection for the LH assay were assigned the lowest measurable value (28/90 samples). Intra- and inter-assay coefficients of variation were 5.3 and 4.9%, respectively. Some samples did not have sufficient plasma to quantify all hormones, thus sample sizes vary.

### Gene Expression

Total RNA was extracted from pituitary samples using an RNAqueous micro kit (AM 1931, Ambion, Life Technologies, Carlsbad, CA) and from placental samples using ISOLATE II RNA Mini-kit (BIO-52073, Bioline United States Inc., Taunton, MA). RNA concentration and purity were assessed by spectrophotometry (NanoDrop 2000; Thermo Fisher Scientific). The RNA quality of a random subset of samples was analyzed on an Agilent Technologies Bioanalyzer and yielded an RNA integrity number (RIN) of 7.6 or higher for pituitaries and 9.4 or higher for placentae. Reverse transcription was performed using Takara Bio PrimeScript RT Reagent Kit with gDNA Eraser (cat. No RR047A, Mountain View, CA) and then frozen at -20°C until qRT-PCR was performed.

Analysis of relative gene expression via qRT-PCR was performed using SSOAdvanced SYBR Green supermix (BIO-RAD, 1725272, Hercules, CA, United States). Samples were run on a BIO-RAD CFX384 machine with 10 µL reaction volumes with a 2-step amplification for 40 cycles with an annealing temperature of 60°C followed by a melt curve. Primers were designed from published sequences for *Mus musculus* using NCBI Primer BLAST software (Supplementary Table S1). Primer sets were validated for specificity using positive, negative, no reverse transcriptase, and no template controls, and confirmed with a single-peak melt curve and correct product length. Efficiency of each primer set was determined by standard curve; primers were 95.3–106.2% efficient with *R* ^2^ values above 0.99. All samples were run in triplicate. Replicate sets in which Cq values varied beyond 0.5 cycles were excluded from analysis and resulting data were analyzed in Microsoft Excel following the ΔΔCq method (Pfaffl, 2001).

The geometric mean for the expression of two housekeeping genes was used for reference where possible. We tested several housekeeping genes across all tissues, and we removed those that varied across treatments based on linear models identical to those used for analyses (see *Statistical Analysis*). *Rplp* and *Tubb* were used as reference genes for the pituitaries, *Rplp* and *TBP* were used as reference genes for the labyrinth zone, and *TBP* was used alone for the junctional zone. In all gene replicate groups, the Cq standard deviations for chosen housekeeping genes were smaller than 0.2. All data are expressed as a fold-change over early-pregnancy, control individuals. Some samples did not have sufficient cDNA to quantify the expression of all genes, thus sample sizes vary.

### Placental Dissection, Histology and Image Analysis

#### Tissue Preparation and Histology

Fresh-frozen placentae were sectioned on a cryostat for gene expression and histological analyses. Placental junctional and labyrinth zones (JZ and LZ, respectively) were separated using punches for RNA extraction and gene expression analysis in 60 µm sections. A 40 µm section of each placenta was collected for PCR-based sexing. For histological assessment, sections were collected at 20 µm from these same placentas. Sections were thaw-mounted on slides and stored at −20°C until staining. Slide-mounted tissue was fixed in 4% PFA for 30 min, stained with hematoxylin and eosin Y, and cleared with HistoClear before coverslipping with Permount (SP15-500, Fisher Scientific).

#### Placental Image Analysis

Placental sections were scanned using on a Zeiss AxioScan.Z1 microscope at the CRL Molecular Imaging Center at the University of California, Berkeley. Images were taken at 5x and individual tiles were automatically stitched together in the Zeiss ZEN two Slidescan application (blue edition) to create a single image of the placenta. Cross-section photos were then converted from proprietary .czi file types to .tiff file types in the Zeiss ZEN 3.1 application (blue edition). Four representative scans from each placenta were selected by an investigator unaware of their experimental condition.

A custom MATLAB script was created to process the selected images and quantify the total cross-section area, LZ area, JZ area, percentage of each zone containing tissue, and length of the LZ-JZ border. For the full script, please refer to our GitHub repository: https://github.com/Kriegsfeld-Lab/Placenta-Morphology-Analysis-2021.

Briefly, the MATLAB script employed a color analysis to divide the placental zones - labyrinth, junctional, and decidua-according to their characteristic colors following H&E staining (See Supplementary Figure S2). Four sections from each placenta were randomly selected for morphological quantification and analysis. First, each original photomicrograph was divided into eight different clusters based on a CMYK color map, controlling for the intensity and contrast of the stain on each tissue section. The user, unaware of treatment assignment, was prompted to choose which of the eight CMYK clusters was the brightest in all placental zones and the script then focused on that cluster for the remainder of the analysis. Next, the chosen CMYK cluster was divided into eight different segmentations based on distance between color peaks on an HSV color map. As the different placental zones absorb hematoxylin and eosin Y differently, each zone is best represented by a different color. The eight largest color peaks on the HSV map were divided such that each zone was most visible in the color segmentation that was closest to the stain color of that zone. This information was used to assign a color segmentation to each zone. The user was then prompted to outline each zone (LZ, JZ, and decidua) on the color segmentation chosen for each zone, respectively. This outline directed the script to the location to be analyzed and the perimeter of each placental zone to be defined. A separate image was generated for each zone (Supplementary Figure S2) and the perimeter outline was used to calculate the area of the region, as well as subtract the area that does not contain tissue. The length of the junction of the LZ and JZ was calculated by detecting where their perimeters overlap. Finally, the images of the zone sub-region perimeters, tissue, the LZ:JZ junction line were compiled.

In some instances where chorionic plate tissue or remnants of the yolk sac were adhered to the placenta, they were excluded from the analysis using an accessory script that used the images and calculations created from the main script to recalculate the final data based on user input. This code is also in the GitHub repository.

### Determination of Sex

Placental sections (40 µm) were incubated overnight at 56°C in 200 µL of proteinase K and lysis buffer diluted at 1:100 (200 mM NaCl, 100 mM Tris (pH 8.5), 5 mM EDTA (pH 8.0), and 0.2 percent SDS). The digested samples were centrifuged at 14,000 g at room temperature for 10 min, and the supernatant was poured off into a tube containing 200 µL of isopropanol and subsequently inverted to precipitate the DNA pellet. After a second round of centrifugation (14,000 g, room temperature, 10 min), the isopropanol was aspirated and the DNA pellet for each sample was diluted with 50 µL of TE Buffer (pH 7.6). The polymerase chain reaction (PCR) designed as a probe for sex genotyping was adapted from Simon James Tunster (2017) to yield a 269 bp product from the X chromosome and a 353 bp product from the Y chromosome (Forward: CAC​CTT​AAG​AAC​AAG​CCA​ATA​CA; Reverse: GGC​TTG​TCC​TGA​AAA​CAT​TTG​G) (Tunster, 2017). This probe was used to detect the two-copy Y-linked Rbm31y and the single-copy X-linked Rbm31x in the DNA samples. PCR was performed using MyTaq 2X Red Mix from Bioline (cat. no BIO-25044, Memphis, TN), and 10 µM of Rbm31x/y forward and reverse primers. Thermocycler conditions were 94°C for 2 min, followed by 30 cycles of 94°C for 20 s, 60°C for 20 s and 72°C for 30 s with a final elongation period of 72°C for 5 min. PCR reaction products were mixed with 6X Blue Gel Loading Dye and then electrophoresed onto a 1.5 percent agarose gel.

### Statistical Analyses

Analyses were conducted in R (R Core Team, 2021) using base R functions as well as packages lme4 (Bates et al., 2015, 4), lmerTest (Kuznetsova et al., 2017), emmeans (Lenth, 2021), and scales (Wickham et al., 2020). For all analyses, *p* < 0.05 was considered statistically significant.

Effects of gestational day and stress on maternal physiological measures (e.g., hypothalamic RFRP-3-ir and TIDA neuron abundance, hormone concentrations, pituitary gene expression) were tested using linear models. Significant main effects were examined via *post hoc* contrasts using Bonferroni-Holm correction for multiple comparisons.

Effects of gestational day and treatment on maternal mass were tested using linear mixed models with dam as a random effect.

Mann Whitney U tests were used to compare litter size and resorption site counts between treatment groups. A logistic regression using the glmer procedure was used to test for effects of treatment on sex bias within litters.

Differences in embryo mass attributable to treatment were assessed using linear mixed models including an interaction between treatment and embryonic sex and dam as a random effect. Litter size had no effect on any models, nor did it differ between treatments and it was thus excluded. Ten (10) embryo masses were excluded from the dataset due to technical errors in dissection.

The effects of stress exposure on placental structure were examined using a series of linear mixed models (See Supplementary Table S2). We summed variables across four representative images of each placenta to reduce bias and noise in analysis due to using a single image. All analyses were conducted on these summed variables. Linear mixed models all included dam as a random effect. We used the rescale function from the scales packages to rescale area measurements for analysis.

Finally, to test for effects of stress exposure on gene expression in the placenta, a series of linear mixed models were used (See Supplementary Tables S3, S4 for full models and results). In all models, dam ID was included as a random effect. Models included fetal mass as a proxy for developmental stage. We used the rescale from the scales packages to rescale gene expression values from qRT-PCR for analysis.

## Results

### Hypothalamic Networks Are Not Affected by Stress

**FIGURE 2:**
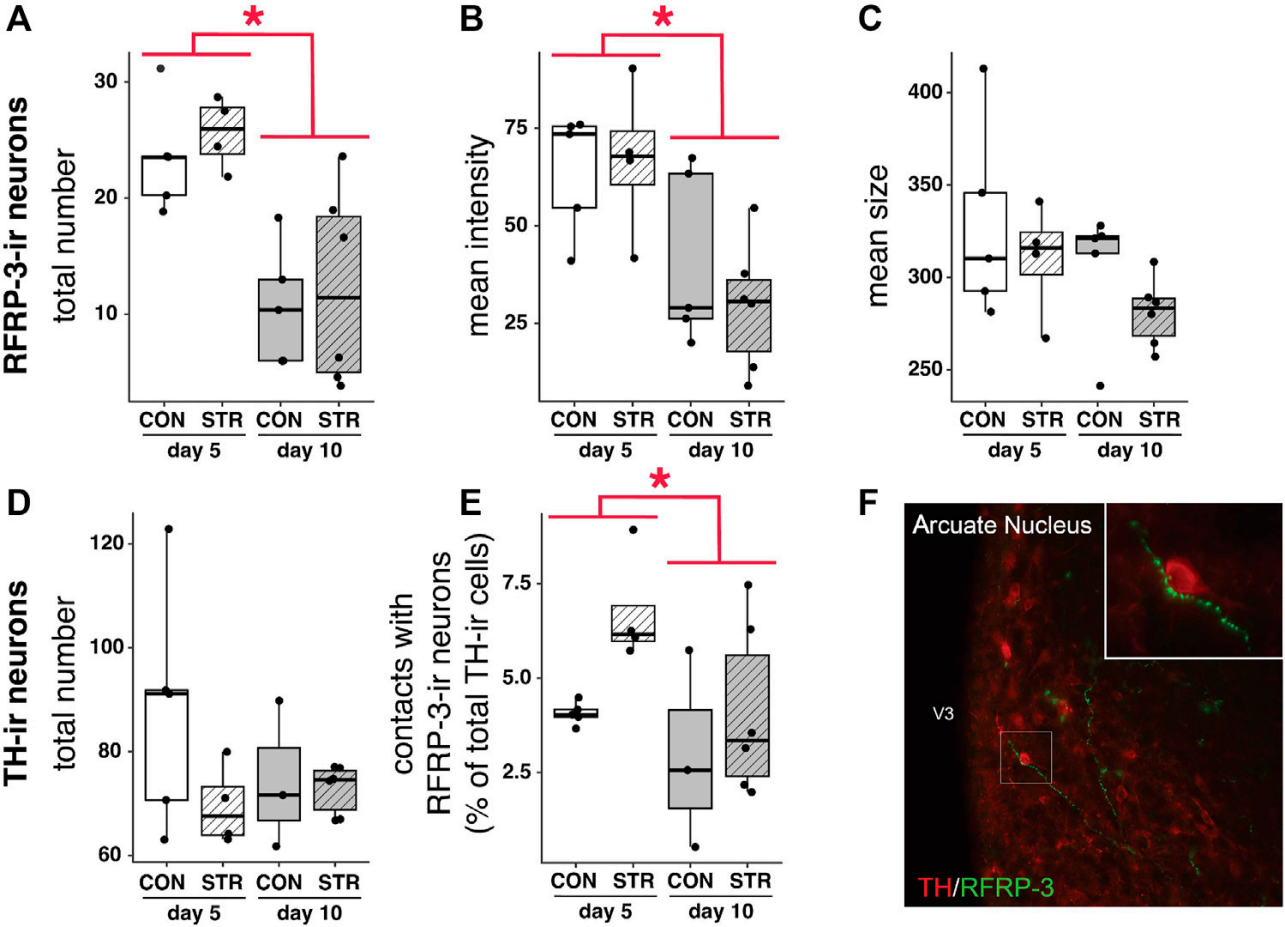
RFRP-3-ir cell numbers/intensity and projections to TIDA (TH+) neurons are impacted by stage of pregnancy, but not by stress. **(A–C)** Number and intensity of RFRP-3-ir neurons were modulated by pregnancy progression but unaffected by stress, whereas the size of RFRP-3-ir neurons remained constant in all treatments. **(D,E)** The number of TH + cells was unaltered by gestational day of pregnancy or stress exposure, whereas contacts between TH + cells and RFRP-3-ir neurons decreased across gestation. **(F)** A representative image of TH + neuron receiving close contacts from RFRP-3-ir axon. High power inset shows contacts in further detail. Asterisks (*) indicate significant (*p* < 0.05) main effects.

The number of RFRP-3-ir cells decreased as pregnancy progressed (Figure 2A; Gestational day: F_1,16_ = 22.69, *p* < 0.0003), as did the intensity of labeling (optical density) in RFRP-3-ir cells (Figure 2B; Gestational day: F_1,16_ = 12.81, *p* < 0.003). There was no significant effect of gestational day or stress on the size of RFRP-3-ir cells (Figure 2C; *p* > 0.11 for all comparisons).

TH-ir cell numbers did not change across gestation nor with chronic stress exposure (Figure 2D, *p* > 0.11 for all main effects). However, gestational day did affect the percentage of TH-ir cells contacted by RFRP-3-ir fibers: the percentage of hypothalamic TH-ir neurons receiving close contacts from RFRP-3-ir axons decreased from early-to mid-gestation (Figure 2E, Gestational day: F_1,14_ = 5.017, *p* < 0.05). Stress did not impact the percentage of contacts between RFRP-ir fibers and TH-ir cells (Figure 2E).

### Pituitary Expression of Prolactin (Prl) mRNA and a Relevant Regulatory Receptor Were Impacted by Stress Early in Pregnancy, but Circulating Prolactin Remained Unaltered

**FIGURE 3:**
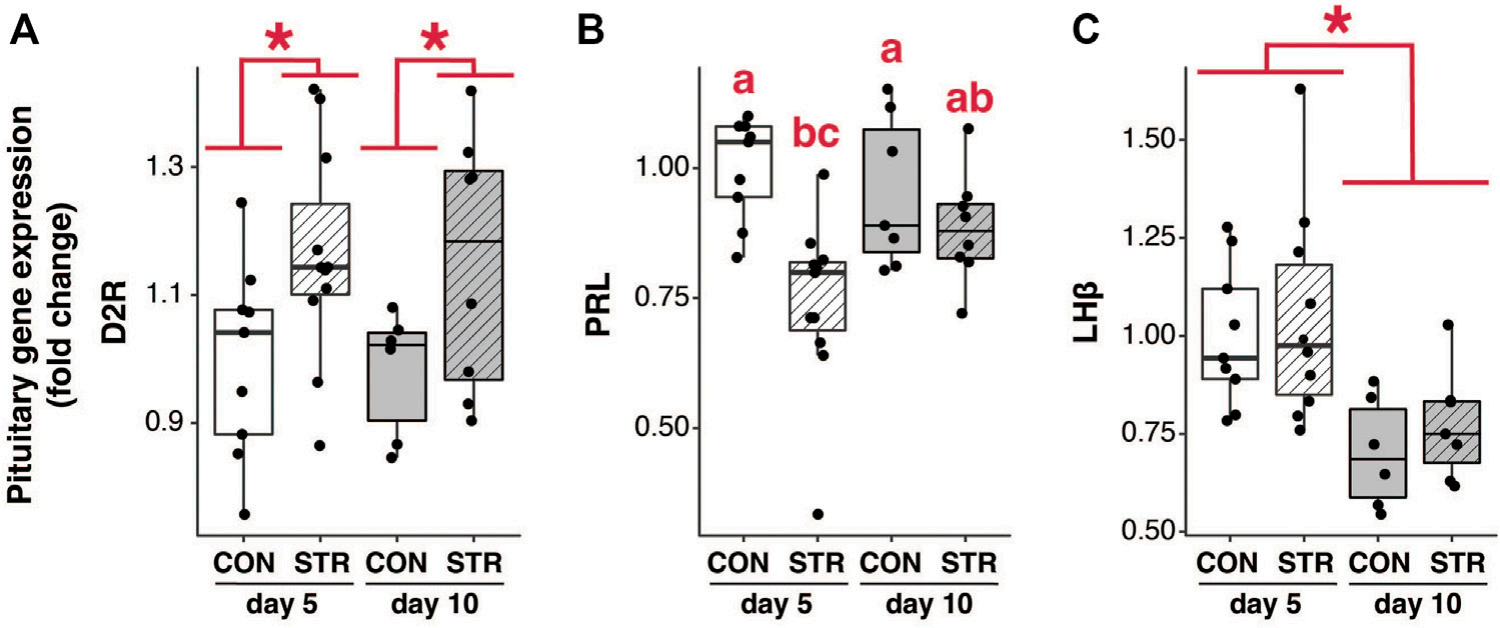
The impact of stress on pituitary mRNA expression. **(A)** Pituitary *D2 receptor* (D2R) mRNA expression was increased in both early- and mid-pregnancy by chronic psychological stress exposure. **(B)** Pituitary *Prl* mRNA expression was attenuated by stress in early-, but not mid-pregnancy. **(C)** pituitary *LHβ* mRNA expression was unaffected by stress, but exhibited a reduction as pregnancy progressed. Asterisks (*) indicate significant (*p* < 0.05) main effects. Different letters indicate significant differences (*p* < 0.05) in *post-hoc* analyses using the Bonferroni-Holm correction for multiple comparisons.

Chronic stress elevated pituitary *D2 receptor* mRNA expression at both early- and mid-gestation (Figure 3A; Treatment: F_1,30_ = 8.50, *p* < 0.007). Chronic stress also resulted in a reduction of *Prl* mRNA abundance during early gestation, however it had no effect on mid-gestation abundance (Figure 3B; Treatment×Gestational Day: F_1,31_ = 4.25, *p* < 0.05).

**FIGURE 4:**
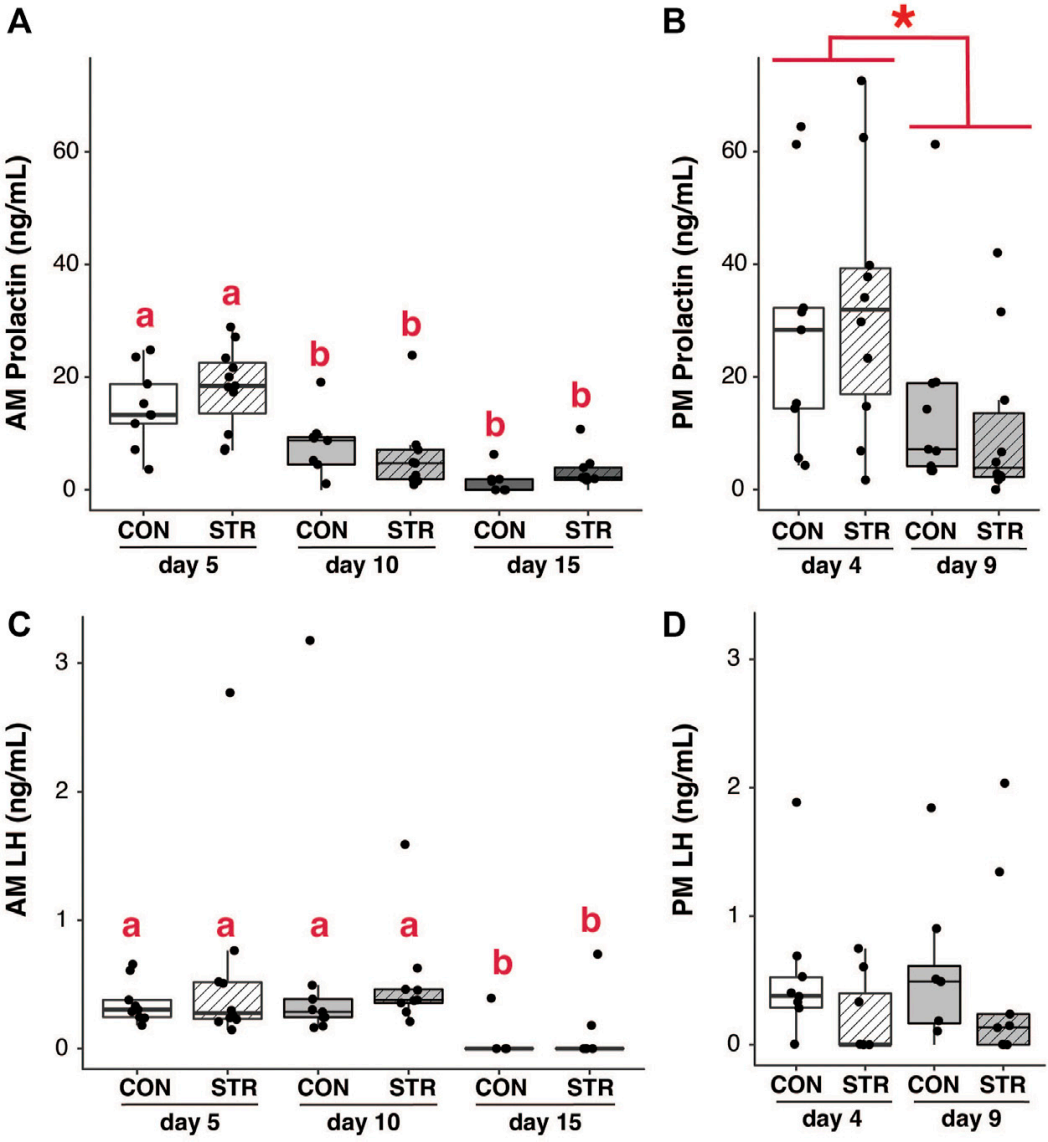
Circulating pituitary hormones change across pregnancy but are unaffected by chronic stress. **(A)** Morning circulating prolactin decreased as pregnancy progressed, but concentrations did not differ between chronically-stressed and non-stressed mice at either pregnancy stage. **(B)** Similar patterns were found in evening levels of circulating prolactin. **(C)** Circulating morning LH was unaffected stress, but decreased during mid-pregnancy (day 15). **(D)** Circulating evening LH did not differ among treatments. Asterisks (*) indicate significant (*p* < 0.05) main effects. Different letters indicate significant differences (*p* < 0.05) in *post-hoc* analyses using the Bonferroni-Holm correction for multiple comparisons.

Changes in pituitary gene expression did not correspond to changes in the circulating level of prolactin. Circulating concentrations of prolactin were assessed in the morning and late afternoon during the expected times of twice-daily prolactin surges. Circulating prolactin decreased as pregnancy progressed for both morning and evening surges (Figures 4A,B; Gestation AM: F_2,50_ = 29.35, *p* < 3.70E-9; Gestation PM: F_1,34_ = 7.55, *p* < 0.01). However, prolactin concentrations did not differ between chronically stressed mice and control groups at any timepoint (Figures 4A,B; *p* > 0.05 for all).

Pituitary expression of *LHβ* subunit decreased as pregnancy progressed, but was not altered by chronic stress (Figure 3C; Gestational Day: F_1,28_ = 15.44, *p* < 0.0006; *p* > 0.05 for other main effects). Circulating LH similarly decreased across gestation (at least in the morning) but was not altered by chronic stress (Figures 4C,D; AM LH: Gestational Day: F_2,50_ = 4.38, *p* < 0.02).

### Concentration of Steroid Hormones in Maternal Circulation Were Impacted by Stress and Pregnancy Stage

**FIGURE 5:**
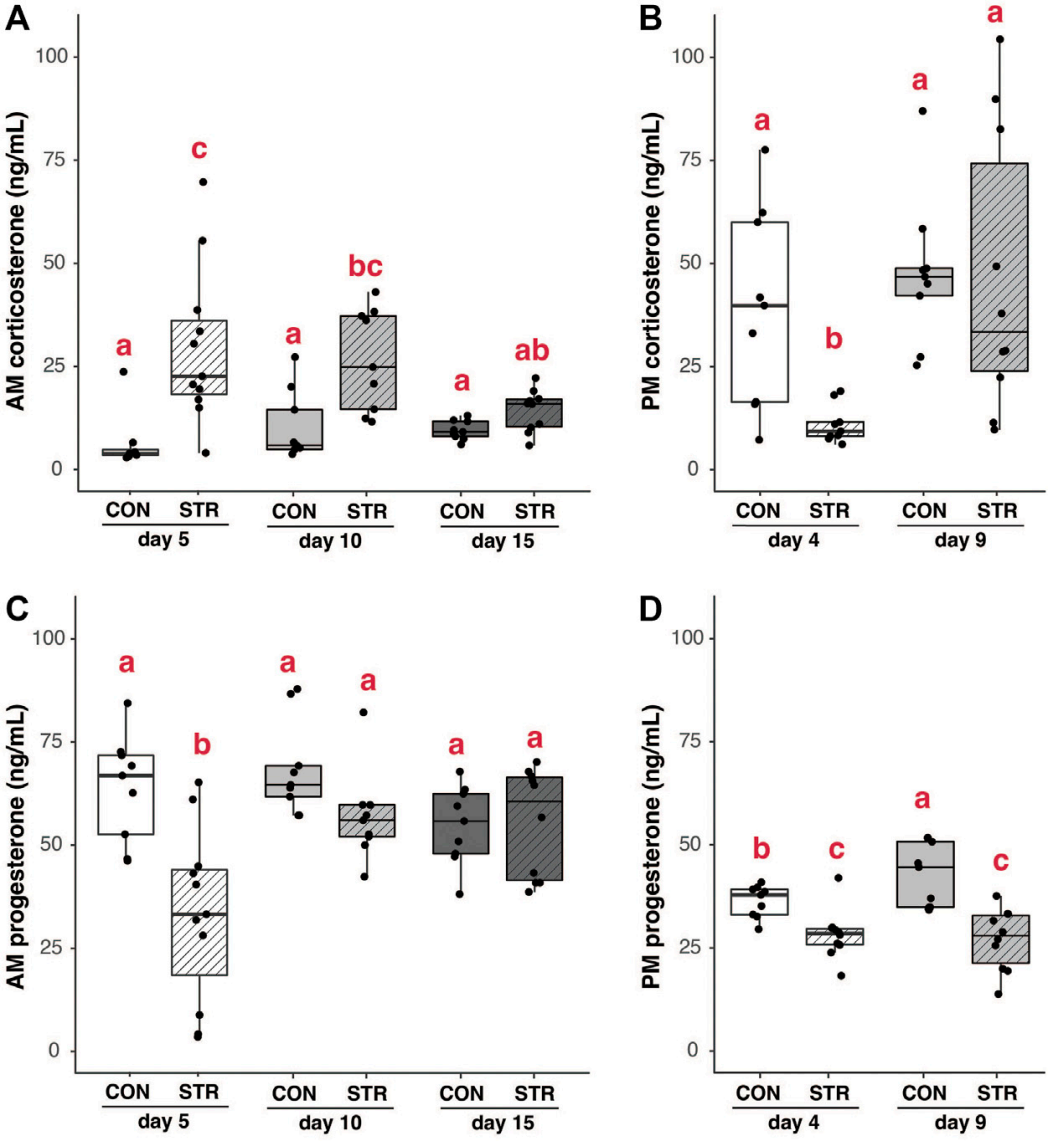
Steroid hormones are modulated by chronic stress and gestation. **(A)** Morning baseline corticosterone concentrations were elevated in chronically-stressed mice on day 5 and 10 of pregnancy, but not on day 15 of pregnancy. **(B)** In the evening, corticosterone was suppressed in chronically stressed dams during early pregnancy (day 5), but unaltered during mid-pregnancy. **(C)** Morning progesterone was lower in chronically stressed dams during early pregnancy, but was unaffected by stress at later timepoints. **(D)** Evening progesterone was lower in chronically stressed dams during both early- and mid-pregnancy compared to non-stressed mice. Different letters indicate significant differences (*p* < 0.05) in post-hoc analyses using the Bonferroni-Holm correction for multiple comparisons.

Chronic stress elevated morning baseline corticosterone concentrations during early and mid-pregnancy relative to unstressed dams (Figure 5A; Treatment×Gestational day: F_2,50_ = 3.37, *p* < 0.04). In the evening, baseline corticosterone was lower in stressed dams during early-pregnancy relative all other timepoints (Figure 5B; Treatment×Gestational day: F_1,33_ = 3.16, *p* = 0.08; *p* < 0.03 for all post-hoc comparisons).

Stress also resulted in the suppression of serum progesterone. In the morning, this suppression was only apparent during early pregnancy (Figure 5C; Treatment×Gestational day: F_2,51_ = 6.11, *p* < 0.005; *p* < 0.004 for all post-hoc comparisons). In the evening, progesterone concentrations were lower in stressed dams regardless of gestational day (Figure 5D; Treatment: F_1,34_ = 32.57, *p* < 2.08E-6). There were no significant correlations between serum corticosterone and progesterone at any time point in pregnancy (all *p* > 0.15).

### Stress Inhibits Maternal Mass Gain Across Gestation

**FIGURE 6:**
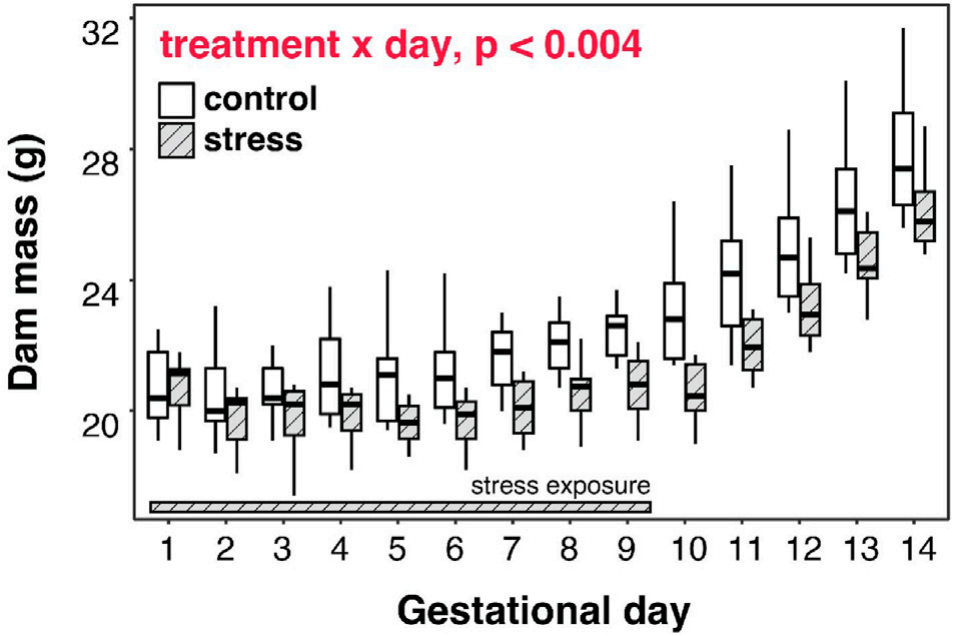
Gestational stress inhibits mass gain by dams, and progesterone is correlated with mid-term mass in stressed dams. Pregnant mice that were exposed to chronic stress for the first 9 days of gestation exhibited a different pattern of mass gain throughout gestation, compared to non-stressed mice, with a magnitude of difference increasing as pregnancy progressed.

Chronically-stressed mice exhibited a different pattern of mass gain throughout gestation, compared to non-stressed mice (Treatment×Gestational day: F_1,245_ = 8.9, *p* < 0.004). Although both groups gained mass across their pregnancies, stressed females exhibited smaller mass gains than non-stressed females across the pregnancy and thus remained lower in average mass throughout gestation (Figure 6). Some of this slower gain in mass was driven by mass loss across the first 6 days of gestation (see Supplementary Figure S3).

### Stress Results in Variable Embryo Development

**FIGURE 7:**
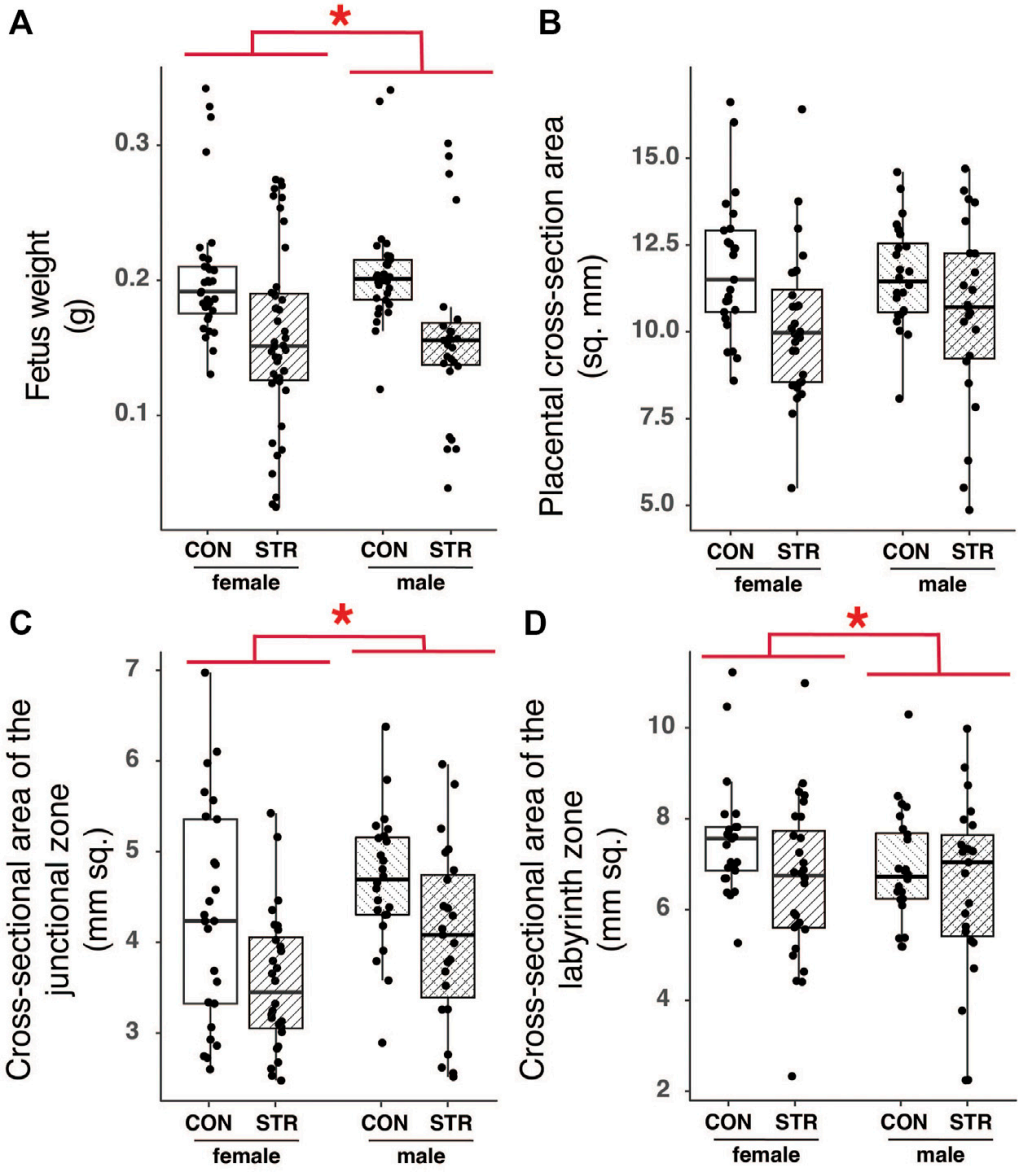
Chronic psychological stress exposure did not affect fetal or placental outcomes. **(A)** Within litters, male embryos tended to be larger than female embryos, but there were no effects of stress on mass. **(B)** Placental cross-sectional areas did not differ by fetal sex or with gestational stress exposure. **(C)** The area of the junctional zone was larger in male embryos compared with female embryos, but it was not altered by gestational stress. **(D)** Female labyrinth zones tended to be larger than that of males, but there was no effect of gestational stress on the area. Letters indicate significant differences (*p* < 0.05) in post-hoc analyses using the Bonferroni-Holm correction for multiple comparisons.

We found no effect of stress on embryonic mass irrespective of sex (Figure 7A; Treatment: F_1,16.95_ = 2.79, *p* = 0.11). However, we also recorded developmental stages for embryos from a subset of dams within the experiment (Control N = 4; Treatment *N* = 6) and we found that while developmental stage was consistent among embryos from non-stressed mice (N = 4; all embryos staged at TS 22 or 23), embryos from chronically stressed mice exhibited a higher variation in developmental stage such that 35% of embryos were at TS < 22 and 50% of dams (*N* = 6) had embryos at TS < 22 (Table 1). Formal analyses of these differences are precluded by sample size. Nonetheless, we also found no effect of stress on litter size or the number of resorption sites per dam (Litter size: *W* = 44, *p* = 0.97; Resorption sites: W = 56, *p* = 0.34; Table 1). Finally, there was no effect of treatment on sex-bias within litters (logistic regression, Treatment: *p* = 0.32).

**TABLE 1:** Descriptive parameters of embryo development in chronically-stressed and non-stressed (control) dams. Pregnant mice were exposed to stress for the first 9 days of gestation, 4 h per day, and allowed six more days to recover before tissue collection at gestational day 15. Non-stressed pregnant mice remained in their home cage.

	Control (*n* = 9)	SEM	Stress (*n* = 10)	SEM
Mean number of embryos	7.78	0.42	7.9	0.34
Mean number of resorption sites	0.89	0.19	0.7	0.28
Mean embryo mass (grams)	0.201	0.01	0.154	0.01
Median embryo mass	0.199	0.01	0.150	0.01
Mean developmental stage	22.63 (*n* = 4)	0.09	20.833 (*n* = 6)	0.42
Median developmental stage	23 (*n* = 4)		21.5 (*n* = 6)	
Percent of embryos < TS22	0 (*n* = 4)		35 (*n* = 6)	
Number of dams with embryos < TS22	0 (*n* = 4)		3 (*n* = 6)	
Female: male ratio	4.2 : 3.5		4.8 : 2.8	

### Gestational Stress did Not Alter Structure or Function of the Placenta

#### Morphology

To examine the long-term impact of chronic stress on placental morphology, we quantified the total area (mm^2^) of the JZ and LZ, tissue density within these regions, and the tortuosity of the junction between the JZ and the LZ (Supplementary Figure S2). Fetal mass, used here as a proxy for developmental stage, had a strong and significant effect on most measures of placental histology; larger fetuses, presumably of later developmental stages, had larger placentas (Sex: F_1,17.66_ = 35.07, *p* < 0.0001). Fetal sex also impacted the make-up of the placenta, with male embryos having larger junctional zones relative to female embryos in both absolute and relative terms (Figure 7C; Supplementary Table S2). Tortuosity of the junction between the junctional and labyrinth zone was similarly unaffected by stress during gestation (see Supplementary Table S2).

#### Gene Expression

Using qRT-PCR, we quantified expression of a panel of genes relevant to placental function and development (see Introduction). Developmental stage had a significant effect on expression of all genes of interest in both junctional and labyrinth zone, except for *Tpbpa* in either zone and *11β-HSD1* in the labyrinth zone (*p* < 0.006 for all; see Supplementary Tables S3, S4). When controlling for developmental stage, there was no effect of stress on expression of genes of interest in either the JZ or LZ (*p* > 0.15 in all cases; see Supplementary Tables S3, S4).

## Discussion

The present study sought to investigate two questions: whether psychological stress during in early pregnancy modifies neuroendocrine circuitry and hormone production, and whether fetal and placental development are impacted by this stressor. Although we found that chronic psychological stress resulted in elevated corticosterone in gestating dams (Figure 5A), demonstrating the stressor activated the HPA-axis, our results suggest that major components of the reproductive neuroendocrine system, the fetus, and the placenta are resilient to psychological stress up until mid-pregnancy. In addition to identifying select neuroendocrine resilience to psychological stress during pregnancy, we identified novel connectivity between RFRP-3-ir and TIDA neurons, and we reveal dynamic regulation of this putative circuit across early gestation. Together, these results pose new questions about the mechanism(s) underlying maternal and embryonic resilience to stress during early to mid-gestation.

In general, our results qualitatively align with previous work on chronic stress in pregnant mice (Wilsterman et al., 2018), finding that stress-related suppression of progesterone release is not associated with changes to the production of the pituitary hormones PRL or LH. Our study also supports the hypothesis that maternal body condition or energy balance contributes to changes in progesterone (Wilsterman et al., 2018). Intriguingly, new data from this study suggest that stress exposure alters the daily pattern of corticosterone production (see Figures 5A,B), perhaps pointing to effects of stress on the circadian system (e.g., Smarr et al., 2017; Varcoe, 2018; Gotlieb et al., 2020). More detailed work on markers of energy balance that may influence progesterone secretion and circadian dynamics related to stress responses during gestation are needed to disentangle the mechanisms driving this relationship.

In this study, we identified novel connections between RFRP-3-ir cells and TIDA neurons (Figure 2). These connections suggest a novel pathway by which stress, via corticosterone action on RFRP-3, might impact prolactin regulation. Surprisingly though, we found no effects of gestational stress on any of the hypothalamic or hormone measures within this putative pathway. The absence of any change in hypothalamic RFRP-3-ir in particular was unexpected because previous work in non-pregnant or pre-pregnant mice and rats consistently showed stress-dependent changes in RFRP-3-ir cell activation and gene expression (Kirby et al., 2009; Geraghty et al., 2015; Yang et al., 2018; Singh et al., 2022). Thus, our study suggests that maternal neuroendocrine systems are buffered from stress during the first two thirds of gestation. Indeed, across mammalian species, including humans, extensive morphological and functional central changes occur during pregnancy (Zingg et al., 1995; Galea et al., 2000; Grattan et al., 2001; Oatridge et al., 2002; Kokay et al., 2006; Pawluski et al., 2009; Hillerer et al., 2014; Kim, 2016), and during late gestation, these changes include an attenuated HPA response to a variety of stressors (Douglas et al., 1998). Because our study focused on early to mid-gestation with dams displaying a robust change in production of glucocorticoids, HPA axis attenuation cannot explain out results, and instead our data point to a novel system responsible for buffering the effects of stress on maternal physiology. Dynamic mechanisms are likely necessary in part because the fetus and placenta increasingly participate in neuroendocrine regulation of maternal physiology during late gestation; thus, maternal physiology must be dynamically regulated as well. More specifically, our study suggests that, during early pregnancy, changes in the connectivity between RFRP-3-ir neurons and other hypothalamic mediators of the stress response (e.g., changes in GR receptor expression) may contribute to stress resilience.

The failure to detect changes in putative RFRP-3-TIDA-mediated prolactin release is surprising given that we found evidence for changes in the mRNA abundance for both *D2r* and *Prl* in the pituitary gland of stressed dams. The pattern of gene expression for *D2r* and *Prl* in the pituitary gland is consistent with stress-dependent increases in the inhibitory tonicity of dopamine on prolactin transcription in the pituitary (Maurer, 1981; Ben-Jonathan and Hnasko, 2001), suggesting that stressed dams were indeed experiencing greater dopamine-dependent inhibition of prolactin production. One possible explanation for the absence of any change in circulating prolactin is that transcriptional changes did not contribute to significant changes in protein production, ultimately equalizing prolactin production and release between treatment groups. Alternatively, there may be a difference in prolactin production that we failed to detect in our study due to the pulsatile pattern of prolactin release during pregnancy. With a pulsatile secretion pattern, a single time point may not be representative of the dynamic changes in hormone secretion (Guillou et al., 2015; Grant et al., 2018). Notably, the surge values measured here are lower than expected (e.g., Barkley et al., 1978; Helena et al., 2009), suggesting we did not capture the peak of the surge in our sampling. Greater temporal resolution hormone measures (e.g., serial/time course sample collection) are needed to determine definitively whether our stress paradigm leads to hypothalamic-pituitary dysregulation and stress-induced hormone dysregulation.

A major motivation for carrying out this study was evidence for a host of stress-related detrimental effects on implantation, litter size, embryo resorption, fetal growth, and survival rates (Arck et al., 1995; Haque et al., 2004; Kondoh et al., 2009; Geraghty et al., 2015; Jafari et al., 2017). However, when controlling for developmental stage, we found no effect of stress on fetal mass. It is possible that differences in mass or growth would have emerged later in gestation, as fetal growth increases exponentially during the final third of gestation. In agreement with this possibility, in other studies that were focused on chronic stress and fetal growth outcomes, no effects on growth or placental phenotypes were detected until near-term (Oatridge et al., 2002). Thus, an absence of stress-related effects on fetal growth at mid-pregnancy does not indicate that chronic psychological stress during gestation is inconsequential. Examining placental and fetal phenotypes across the final third of pregnancy under similar stress protocols as used here will be informative for understanding how and when late gestation phenotypes emerge.

Despite observing no effect of stress on fetal mass, we found substantial variability in litter development among dams subjected to psychological stress that was absent in control dams. Variability in litter development among stressed dams indicates individual variation in sensitivity or susceptibility to the chronic stress applied in this study. Individual differences in stress response are surprising given that the mice in this study are genetically identical. Differences in vulnerability and resilience may arise from epigenetic modifications linked to early life experience or social position. For example, in humans, methylation in the promoter of the GR NR3C1 is associated with maternal prenatal stress (Radtke et al., 2011; Mulligan et al., 2012; Hompes et al., 2013). Social factors prior to pregnancy, including whether dams were dominant or subordinate, could also contribute to epigenetic variation or susceptibility [e.g., (Nesher et al., 2013; Gross and Pinhasov, 2016; Gross et al., 2018; Bairachnaya et al., 2019; Murlanova et al., 2021)]. Finally, as alluded to earlier, variation in maternal condition might also contribute to or predict fetal growth trajectories and developmental delays [e.g. (Cross et al., 1994; Spencer and Bazer, 2002; Miller et al., 2004; Geraghty et al., 2015)]. Clarifying the interactions and mechanisms that lead some individuals to be resilient versus susceptible to stress will be important for explaining individual variation in outcomes, as seen here.

Overall, the present findings suggest that the impact of stress on glucocorticoid production is evident well past its cessation, but that many aspects of reproductive function are largely unaffected up until mid-gestation. Nonetheless, variation in litter development among stress-exposed-mothers points to the importance of understanding individual variation in susceptibility to stress in terms of predicting risk for adverse gestational outcomes. Finally, the impact of stress during pregnancy appears to occur via mechanisms distinct from those seen in non-pregnant animals. Further studies examining these alternative pathways at different stages of pregnancy are important for understanding how stress at different periods of gestation might translate into altered gestational outcomes.

## Data Availability

The datasets presented in this study can be found in online repositories. The names of the repository/repositories and accession number(s) can be found below: https://github.com/kwilsterman/ChronicPrenatalStress_2022.git.
