# Supplementary figures and images for: Impact of Chronic Prenatal Stress on Maternal Neuroendocrine Function and Embryo and Placenta Development During Early-to-Mid-Pregnancy in Mice
Source: Front Physiol. 2022 Jun 13;13:886298. doi: 10.3389/fphys.2022.886298 (PMC9234491; doi:10.3389/fphys.2022.886298)

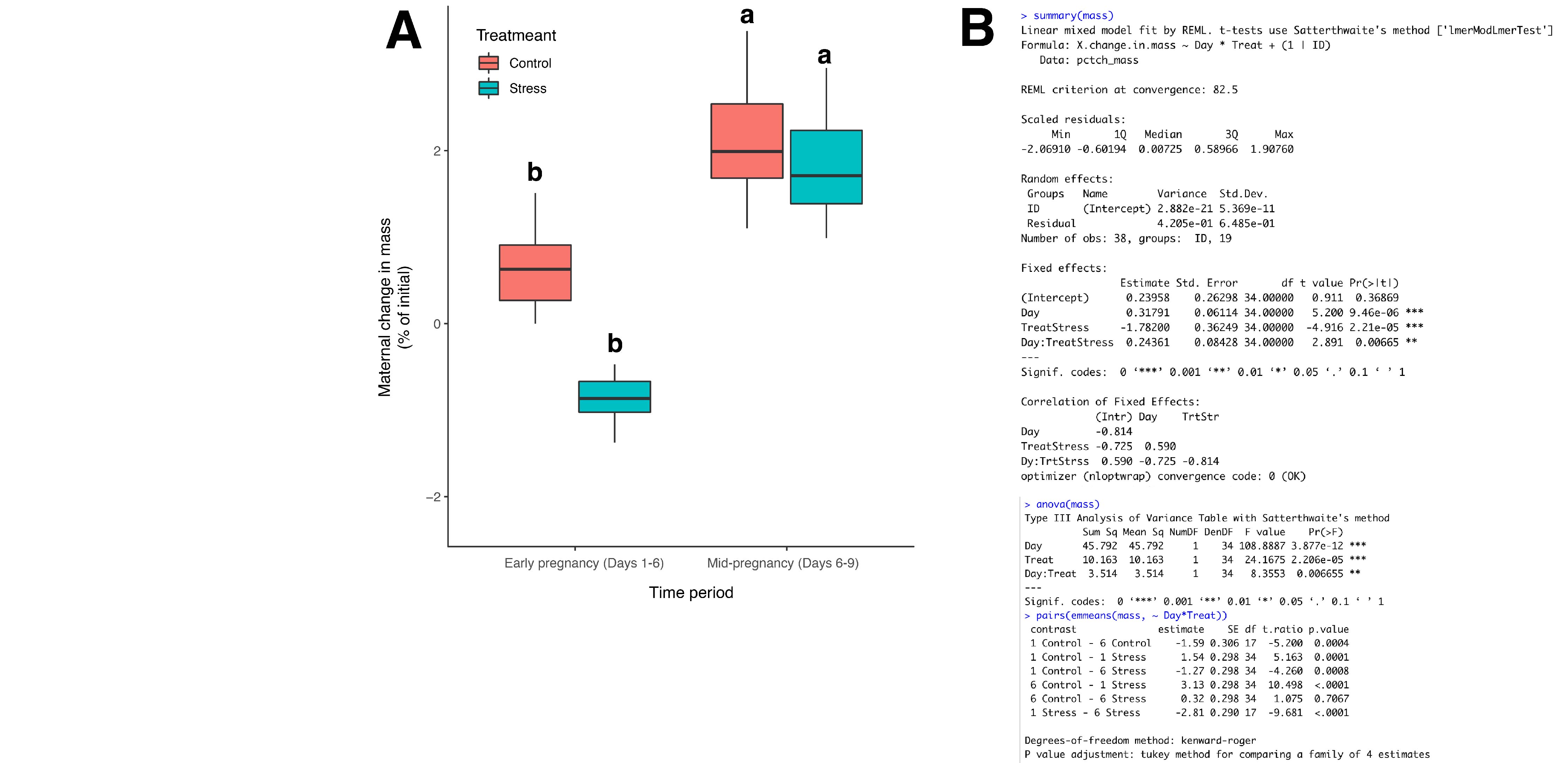

Supplement: Supplementary file 1 [file Image3.JPEG]

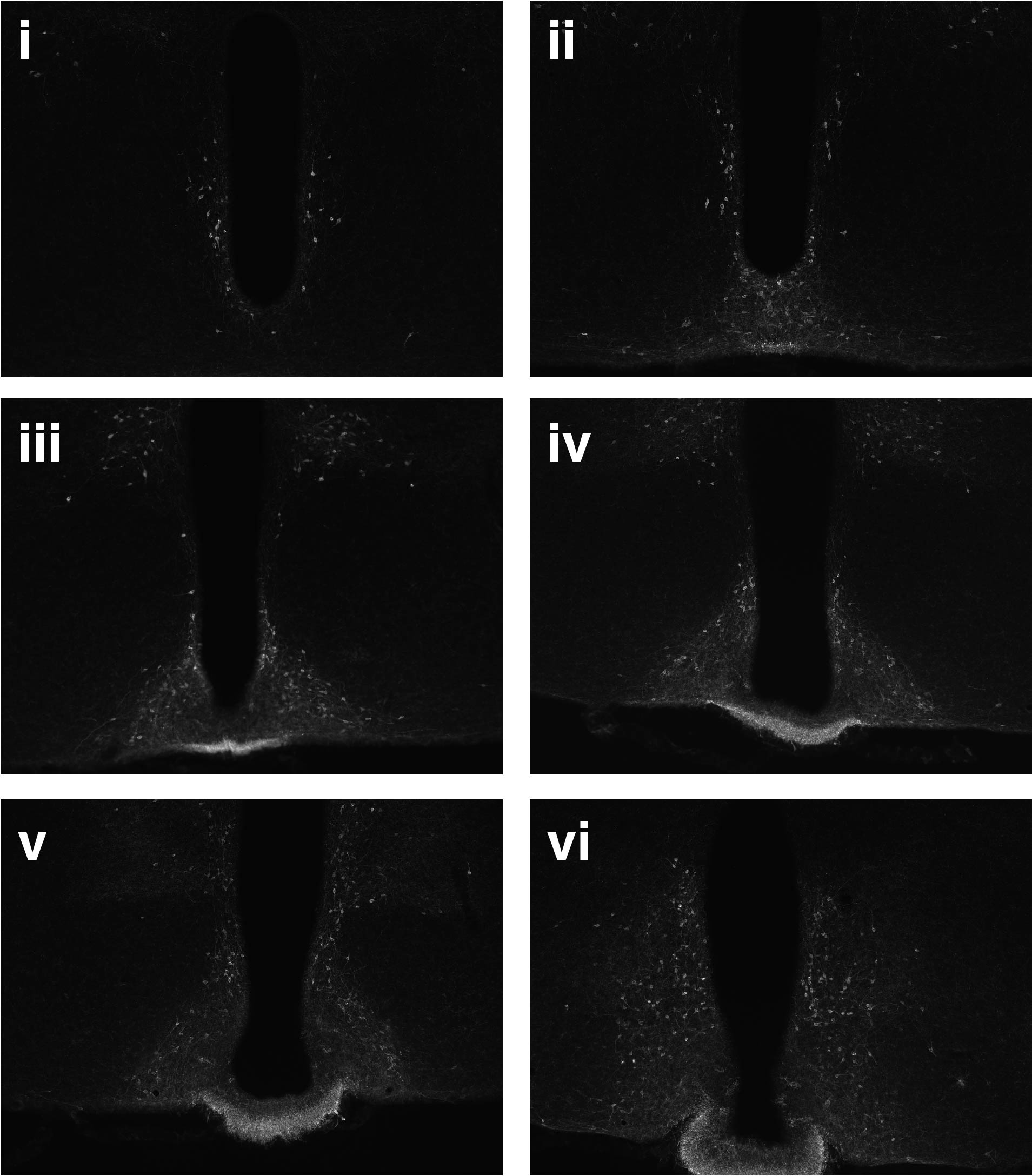

Supplement: Supplementary file 2 [file Image1.JPEG]

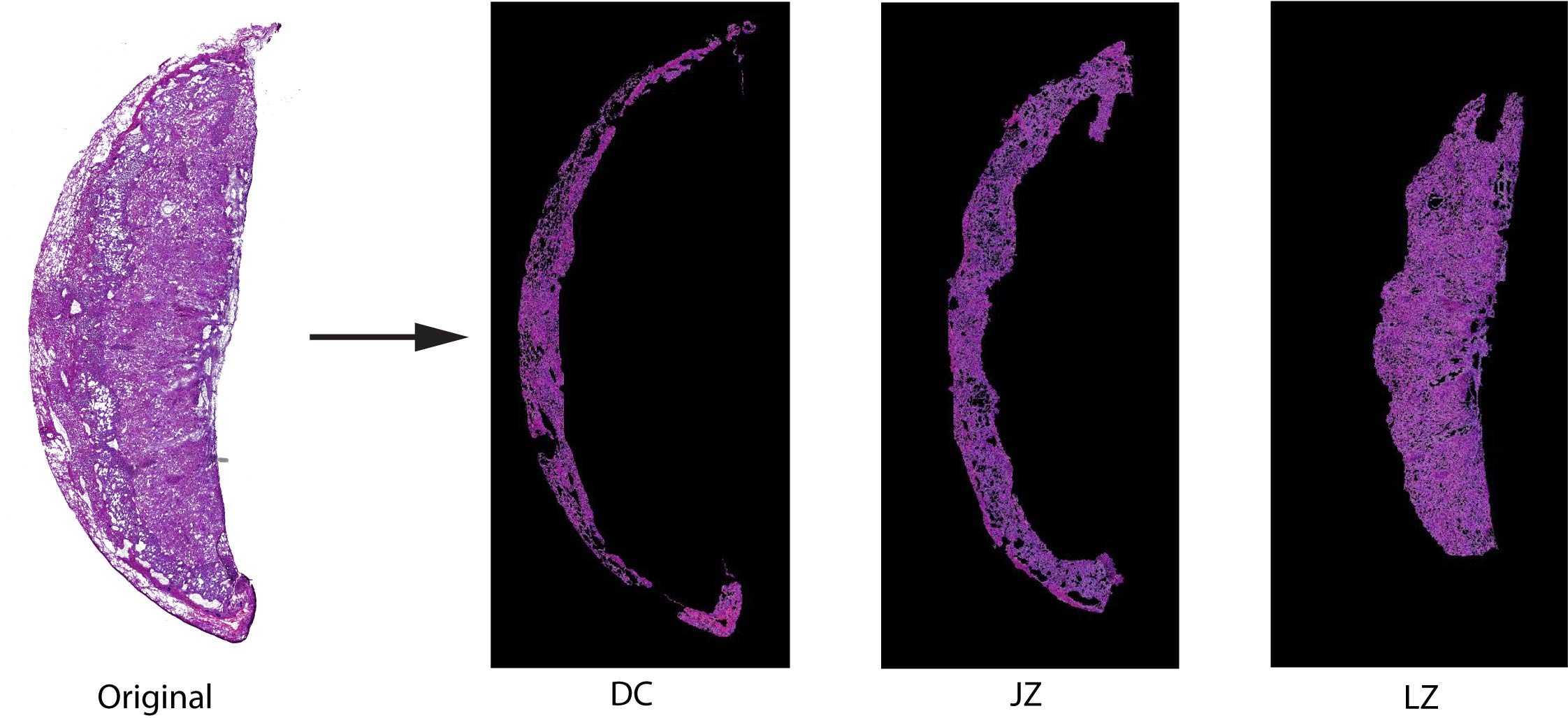

Supplement: Supplementary file 3 [file Image2.JPEG]
